# RhoGDI1-Cdc42 Signaling Is Required for PDGF-BB-Induced Phenotypic Transformation of Vascular Smooth Muscle Cells and Neointima Formation

**DOI:** 10.3390/biomedicines9091169

**Published:** 2021-09-06

**Authors:** Yan Qi, Xiuying Liang, Haijing Guan, Jingwen Sun, Wenjuan Yao

**Affiliations:** Department of Pharmacology, School of Pharmacy, Nantong University, 19 Qixiu Road, Nantong 226001, China; 1923310019@stmail.ntu.edu.cn (Y.Q.); 17170018@stmail.ntu.edu.cn (X.L.); 1627012011@stmail.ntu.edu.cn (H.G.); 1727011021@stmail.ntu.edu.cn (J.S.)

**Keywords:** RhoGDI, Rho GTPases, VSMC phenotypic transformation, intimal hyperplasia

## Abstract

RhoGTPase is involved in PDGF-BB-mediated VSMC phenotypic modulation. RhoGDIs are key factors in regulating RhoGTPase activation. In the present study, we investigated the regulatory effect of RhoGDI1 on the activation of RhoGTPase in VSMC transformation and neointima formation. Western blot and co-immunoprecipitation assays showed that the PDGF receptor inhibition by crenolanib promoted RhoGDI1 polyubiquitination and degradation. Inhibition of RhoGDI1 degradation via MG132 reversed the decrease in VSMC phenotypic transformation. In addition, RhoGDI1 knockdown significantly inhibited VSMC phenotypic transformation and neointima formation in vitro and in vivo. These results suggest that PDGF-BB promotes RhoGDI1 stability via the PDGF receptor and induces the VSMC synthetic phenotype. The co-immunoprecipitation assay showed that PDGF-BB enhanced the interaction of RhoGDI1 with Cdc42 and promoted the activation of Cdc42; these enhancements were blocked by crenolanib and RhoGDI1 knockdown. Moreover, RhoGDI1 knockdown and crenolanib pretreatment prevented the localization of Cdc42 to the plasma membrane (PM) to activate and improve the accumulation of Cdc42 on endoplasmic reticulum (ER). Furthermore, Cdc42 inhibition or suppression significantly reduced VSMC phenotypic transformation and neointima formation in vitro and in vivo. This study revealed the novel mechanism by which RhoGDI1 stability promotes the RhoGDI1-Cdc42 interaction and Cdc42 activation, thereby affecting VSMC phenotypic transformation and neointima formation.

## 1. Introduction

The arterial response to injury appears to be an important factor in the development of pathological intimal hyperplasia and restenosis. Restenosis after vascular injury remains the major problem in limiting the long-term efficacy of vascular surgery and stenting [1,2], but the mechanisms responsible for intimal hyperplasia and restenosis remain unclear. Vascular smooth muscle cells (VSMCs) play an important role in regulating vascular contraction and dilation. These cells do not terminally differentiate and can undergo a transition between a contractile phenotype and a proliferative synthetic phenotype in response to various pathological stimuli; this transition or dedifferentiation is a key step in the vascular reparative response to injury and atherosclerotic lesion formation or other cardiovascular diseases [3]. Fully differentiated VSMCs highly express contractile proteins, including smooth muscle α-actin (ACTA2), smooth muscle myosin heavy chain (MYH11), transgelin (TAGLN; alternative name SM22), and smoothelin [4,5,6,7]. Proliferation and migration are important manifestations of the VSMC synthetic phenotype. A variety of growth factors, cytokines, and active peptides participate in the process of VSMC proliferation and migration through various signaling pathways [8,9]. Platelet-derived growth factor-BB (PDGF-BB) is a well-known factor driving the processes of VSMC proliferation and migration, which are involved in vascular neointima formation [10]. PDGF activates the RhoA/ROCK, ERK-1/2, JNK, and PI3K/Akt pathways, and it results in cell proliferation and migration [3,11,12,13]. However, the precise molecular mechanisms by which PDGF regulates VSMC phenotypic transformation remain largely unknown.

Rho specific guanine nucleotide dissociation inhibitor 1 (RhoGDI1) —also known as RhoGDIα or ARHGDIA —is critical for the homeostasis of Rho proteins, and it is ubiquitously expressed [14,15]. Many studies have shown that RhoGDI1 expression plays a crucial role in various cellular functions, such as proliferation and migration, as well as the biological behaviors of tumor cells [15,16,17,18,19,20]. Although different studies have reported a relationship between RhoGDI1 and a variety of cancers, reports on the roles of RhoGDI1 in VSMC phenotypic transformation are limited. We have recently revealed that RhoGDI1 participates in VSMC proliferation, induced by renin angiotensin system (RAS) activation [21]; however, the signaling pathway acting downstream of RhoGDI1 to mediate VSMC phenotypic modulation and neointima formation is still unclear.

Rho GTPases belong to the Ras superfamily and can be divided into eight subfamilies, including the well-studied Rho, Rac1 and Cdc42 [22]. Rho GTPases play crucial roles in cell proliferation, apoptosis, gene expression (e.g., cyclin-dependent kinase 5 and preproendothelin-1), and multiple other common cellular functions (e.g., differentiation, adhesion, and secretion) [23,24]. Most Rho GTPases are molecular switches cycling between an inactive guanine nucleotide diphosphate (GDP)-bound state and an active guanine nucleotide triphosphate (GTP)-bound state [25]. Many studies have highlighted the importance of GDI proteins in the regulation of protein expression levels and the activation state of Rho GTPases. Depletion of RhoGDI1 or its yeast ortholog RDI1 results in almost complete degradation of cytoplasmic Rho GTPases by proteasomes and reduced Rho GTPase activity [15]. Furthermore, Rho proteins may fail to properly localize to the plasma membrane (PM) and may remain in the endoplasmic reticulum (ER) without RhoGDI1 [15]. These studies have suggested that one of the functions of RhoGDI1 is to shuttle Rho GTPases from the ER to their site of action at the PM. However, the exact effects of RhoGDI1-Rho GTPase signaling on PDGF-BB-mediated VSMC phenotypic modulation and pathological neointima formation remain unclear.

In the present study, we used an in vitro PDGF-BB-induced HA-VSMC phenotypic transformation model and an in vivo balloon injury-induced neointima formation model to investigate the effects of the RhoGDI1-Rho GTPase pathway on VSMC phenotypic modulation and the regulatory effect of RhoGDI1 on Rac1 or Cdc42 activation.

## 2. Materials and Methods

### 2.1. Materials

PDGF-BB (Cat. No. 100- 14B) and a Fogarty 2F balloon catheter (#12TLW804 F) were bought from PeproTech Inc (Rocky Hill, NJ, USA) and Baxter Healthcare Corp. (Irvine, CA, USA), respectively. Fetal bovine serum (FBS; # F2442) and 4′, 6- diamidino-2-phenylindole (DAPI; # 28718-90-3) were bought from Sigma Chemical Co. (St. Louis, MO, USA). Crenolanib (PDGF-BB receptor inhibitor; # CP-868596), NSC23766 (Rac1 inhibitor; # 587841-73-4), and ZCL278 (Cdc42 inhibitor; # 1177865-17-6) were from ApexBio Technology (Houston, TX, USA) and ChemCatch Co., Ltd. (Shanghai, China), respectively. The primary antibodies against GAPDH (#5174) and β-tubulin (# AT0003) were purchased from Cell Signaling Technology (Beverly, MA, USA) and CMCTAG Inc (San Diego, CA, USA), respectively. Anti-RhoGDI1 (A1214), –Ubiquitin (A3207), –smoothelin (# 6745), –MYH11 (# 10827), -ki-67 (A11390), and -SM22α (# A6760) antibodies were purchased from ABclonal (Wuhan, China). –Anti-Cdc42 (# AF2794) antibody and crystal violet (# C0121) were purchased from Beyotime Technology (Shanghai, China). Anti-Rac-1 (# 24072-1-AP) antibody, peroxidase-conjugated AffiniPure Goat Anti-Rabbit IgG (H + L) (SA00001-2), Cy3-conjugated AffiniPure Goat Anti-Mouse IgG (H + L) (SA-00009–1), and fluorescein (FITC)-conjugated AffiniPure Donkey Anti-Rabbit IgG (H + L) (D110051) were purchased from Proteintech (Chicago, IL, USA) and BBI Life Sciences (Hong Kong, China). Anti-KDEL (ADI-SPA-827-D) antibody was purchased from Enzo Life Sciences (Farmingdale, NY, USA). MG132 (proteasome inhibitor; #S2619), Anti-CD31 (ab24590), and -α-SMA (ab134964) antibodies were purchased from Abcam (Cambridge, MA, USA). CellLight™ EdU Apollo^®^ 567 In Vitro Imaging Kits (100T) (C10310-1) were purchased from RiboBio Co. Ltd. (Guangzhou, Guangdong, China). Protein A/G PLUS-Agarose (#SC-223) and BCA Protein Assay Kit (CW0014S) were purchased from Santa Cruz Biotechnology (Santa Cruz, CA, USA) and CWBiotech (Beijing, China), respectively. MMC (mitomycin C) (#YZ-1444707) and Transwell^®^ systems (8.0 mm pore size) (#3422) were purchased from Solarbio (Shanghai, China) and Corning, Inc. (Corning, NY, USA), respectively. Rac1/Rho/Cdc42 GTPase activity assay kit (# BK030), RevertAid First Strand cDNA Synthesis Kit (#K1622), and Dream Taq PCR Master Mix (#K1071) were purchased from Cytoskeleton, Inc (Denver, CO, USA) and Thermo Scientific (Shanghai, China). siRNAs and primers were synthesized by Biomics Biotechnologies (Nantong, Jiangsu, China). Human smoothelin Alexa fluor 647-conjugated antibody (IC-8278R) was purchased from R&D Systems (Minneapolis, MN, USA). Maxima SYBR Green qPCR Master Mix was from Fermentas (Burlington, ON, Canada). Other chemicals used in this study were of analytical grade and were made in China.

### 2.2. Cell Culture and Treatment

Human aortic VSMCs (HA-VSMCs) were purchased from the American Type Culture Collection (ATCC number: CRL- 1999; Manassas, VA, USA). The cells were cultured in Ham’s F12 K medium containing 2 mM of L-glutamine supplemented with 10% FBS at 37 °C in a humidified 5% CO_2_ incubator. The cells were used at passages 3–7 and treated with 10 ng/mL of PDGF-BB for 24 h. In addition, prior to PDGF-BB treatment, HA-VSMCs were pretreated with crenolanib (50 nM) for 48 h, with MG132 (5 μM) for 1 h, and with NSC23766 (50 μM) or ZCL278 (50 μM) for 30 min.

### 2.3. Rat BI Model

Male Sprague-Dawley rats within the age range of 40-46 days and weighing approximately 250 g were obtained from the Animal Center of Nantong University (Nantong, China). All of the procedures were approved by the Animal Care and Use Committee of Nantong University (15 Apr 2020) (Ethic Committee approval number: 1213201.1) and conformed to the NIH Guide for the Care and Use of Laboratory Animals. Animals were housed in a pathogen-free, temperature-controlled room (22 °C ± 1 °C) with a cycle of light for 12 h and dark for 12 h, and they had free access to food and water. Forty male Sprague-Dawley rats were randomly assigned into the following four groups (*n* = 10/group): sham-operation, model (balloon injury), Ad-RhoGDI1-shRNA-treated group, and Ad-Cdc42-shRNA-treated group. The adenoviral vectors specific to RhoGDI1 and Cdc42 shRNA were purchased from Hanheng Biotechnology (Shanghai, China). RhoGDI1-shRNA: GCAAGATTGACAAGACTGACTATTCAAGAGATAGTCAGTCTTGTCAATCTTGTTTTTT. Cdc42-shRNA: CACCGCTTGTTGGGACTCAAATTGACGAATCAATTTGAGTCCCAACAAGCTTTTTT. Rat carotid artery balloon injury (BI) was performed as previously described [26]. After the BI operation, the carotid artery was injected with approximately 0.2 mL of adenovirus solution for specifically knocking down RhoGDI1 or Cdc42 (titer: 1.0 × 10^10^ pfu) using a small needle. After fourteen days, the rats were euthanized with CO_2_ at a rate of 10–30% infusion per minute. The rats were subsequently immobile, did not breathe, and had dilated pupils. Then the CO_2_ was turned off, and the rats were observed for another 2 min to confirm that they were dead. The BI segment of the artery was removed, washed in ice-cold saline, and used in subsequent experiments.

### 2.4. Western Blot Analysis

After treatment, the cells were lysed in RIPA lysis buffer supplemented with protease and phosphatase inhibitor for 40 min, and then they were centrifuged at 4 °C for 15 min. For tissue protein extraction, the arteries were added to the homogenate beads, and 160 μL of lysis buffer (1% NP-40, 1 mM of PMSF) was added. Protein concentrations were measured using Bradford assays [3]. Equal amounts of proteins were prepared for western blotting. The protein samples were separated by 6–12% SDS-polyacrylamide gel electrophoresis (PAGE) and electrotransferred onto nitrocellulose membranes. The membranes were then blocked with 5% fat free milk for 2 h at room temperature (RT) and incubated with primary antibodies overnight at 4 °C. Antibody-antigen binding was checked by incubation with HRP-conjugated secondary antibodies for 1 h at RT followed by the use of an ECL detection system (Amersham Biosciences; Uppsala, Sweden). GAPDH and β-tubulin were used as internal standards. The relative intensities of the signals were quantified using densitometry and imaging software (Labworks, Maryland, MD, USA).

### 2.5. Real-Time RT-PCR

Total RNA was isolated using a total RNA purification kit (Beyotime Technolog, Shanghai, China), in accordance with the manufacturer’s instructions. One microgram of RNA was reverse transcribed using a first-strand cDNA synthesis kit. The resulting cDNA was mixed with Maxima SYBR Green qPCR Master Mix and used as a template for PCR with a RhoGDI1 specific primer [21]. The amplification conditions used for PCR cycling were as follows: 94 °C for 30 s, 57 °C for 30 s, and 72 °C for 30 s (RhoGDI1); 94 °C for 30 s, 54 °C for 30 s, and 72 °C for 30 s (GAPDH). The products were analyzed by agarose gel electrophoresis after 30 cycles. Quantitative PCR was performed using a Corbett RG-6000 real-time PCR system (Corbett Life Sciences, Mortlake, NSW, Australia). Relative expression levels were determined after normalization to GAPDH. The 2^−ΔΔCt^ method was applied to calculate the relative expression level of RhoGDI1.

### 2.6. Co-Immunoprecipitation (Co-IP)

For the Co-IP assay, the cells from each sample were washed with PBS and lysed with a lysis buffer [50 mM of Tris (pH 7.4), 150 mM of NaCl, 1% Triton X-100, 1% sodium deoxycholate, 0.1% SDS] containing 1 mM of PMSF. The total cell lysates were centrifuged for 15 min at 12,000 rpm to remove insoluble debris after incubation on ice for 20 min. The supernatants were transferred into new tubes, and protein concentrations were determined using a BCA Protein Assay Kit. The lysates were incubated with a RhoGDI1 antibody (1:100) overnight at 4 °C and then with Protein A/G PLUS-agarose at RT for 2 h. Subsequently, the mixture was washed once with normal washing buffer and five times with high-salt washing buffer [21]. Pulled down proteins were boiled at 95 °C for 5 min in SDS loading buffer and analyzed by western blotting. For the ubiquitylation assay, cells were treated with crenolanib or PDGF-BB, together with MG132 before collection. IgG was used as a negative control.

### 2.7. EdU Assay

Cell proliferation was measured using Cell-Light™ EdU Apollo^®^ 567 In Vitro Imaging (100T) Proliferation Assay kits, in accordance with the manufacturer’s instructions [27]. Briefly, cells were seeded into 96-well plates and transferred to serum-free medium for serum starvation for 24 h prior to the indicated treatment. Then the cells were incubated in 100 μL medium containing 50 μM EdU for 2 h. After labeling, the cells were fixed with 4% paraformaldehyde for 30 min followed by permeabilization with 0.5% Triton X-100 in PBS for 10 min at RT. Subsequently, the cells were stained with Apollo and Hoechst 33342 in the dark at RT. After staining, the cells were washed thrice with 0.5% Triton X-100 in PBS. Images were captured by fluorescence microscopy (Nikon, Tokyo, Japan). The experiments were repeated thrice, each using duplicate wells.

### 2.8. Wound Healing and Transwell Assays

The wound-healing assay was performed to check cell migration. HA-VSMCs were grown to appropriate confluence (70%) in 24-well plates, wounded with a sterile pipette tip after different experimental treatments, and then washed with PBS to remove detached cells from the plates. Subsequently, the cells were treated with 10 μg/mL of MMC for 1 h to inhibit proliferation. Cell migration was induced with 10 ng/mL of PDGF-BB for 24 h. Migratory cells from the scratched boundary were counted and averaged from the resulting four phase images for each point under a light microscope (Olympus, Tokyo, Japan). The data were generated from three independent experiments.

Migration of HA-VSMCs was also assessed using a Transwell system with a coated polycarbonate membrane (8.0 μm pore size; Corning). Briefly, 1.0 × 10^5^ cells were seeded in the upper chamber (precoated for invasion) containing 200 μL serum-free medium. Complete culture medium (500 μL) with 10% FBS was added to the lower chamber. Cell migration was induced by 10 ng/mL of PDGF-BB. After incubation for 24 h, the lower chambers were fixed with 4% polymethanol for 30 min and then were stained with 0.1% crystal violet for 30 min. A cotton swab was used to remove the cells from the upper surface of the filter manually. Cells that migrated to or invaded the reverse side of the upper chamber were photographed. Five random fields were selected to calculate the number of cells that had migrated to or invaded the wounded area.

### 2.9. siRNA Transfection

RhoGDI1 siRNA sequence was as follows: sense, 5′-CUUUCCGGGUUAACCGAGAdTdT-3′; antisense, 5′-UCUCGGUUAACCCGGAAAGdTdT-3′. Cells were transfected using Lipofectamine 2000 (Beyotime Technolog, Shanghai, China) in accordance with the manufacturer’s instructions and our previous research (siRNA transfection rate 85%) [21]. Subsequently, the cells transfected with siRNA (knockdown efficiency, 70%) were treated with 10 ng/mL of PDGF-BB for 24 h.

### 2.10. Pulldown Assay

Cells were seeded at a density of 5.0 × 10^4^ cells/mL and grown for 3–5 days. Serum starvation or treatments were performed when the cells reached approximately 30% confluence. Immediately, the cells were rinsed with an appropriate volume of ice-cold PBS and incubated with lysis buffer containing 1 × protease inhibitor cocktail on ice. The protein samples were checked by a pulldown assay in line with the manufacturer’s instructions and our previous research [28].

### 2.11. Immunofluorescence

Sections were fixed with 4% paraformaldehyde (Sangon Biotechnolog, Shanghai, China), quenched with 5% BSA blocking buffer at 37 °C for 30 min, and incubated with primary antibodies (1:100 dilution in PBS) at 4 °C overnight. After washing with PBS, the sections were incubated with FITC-conjugated anti-rabbit IgG for an additional 30 min at 37 °C. The sections were stained with DAPI for 20 min and visualized with a fluorescence microscope (Nikon, Tokyo, Japan). Fluorescence intensity was quantified using ImagePro Plus software (Media Cybernetics, Maryland, MD, USA).

HA-VSMCs were grown on sterile glass coverslips overnight and washed with an appropriate amount of solution, such as HBSS with Ca^2+^ and Mg^2+^. Next, the cells were washed with PBS, fixed with 4% paraformaldehyde for 20 min, and blocked with freshly prepared conventional goat serum at RT for 1 h. The cells were then incubated with primary antibodies (mouse anti-KDEL and rabbit anti-Rac1 or Cdc42 antibodies) overnight at 4 °C. The next day, the cells were incubated with FITC-conjugated donkey anti-rabbit IgG and Cy3-conjugated goat Anti-mouse IgG for 1 h in the dark at RT and then stained with 0.5 μg/mL of DAPI for 20 min. Stained cells were washed and examined under a fluorescence microscope (Nikon, Tokyo, Japan).

### 2.12. HE Staining

The injured segments of the carotid arteries were isolated and fixed in 4% paraformaldehyde and then embedded in optimal cutting temperature (OCT) compound. The embedded surface was trimmed with a cryomicrotome and then cryosectioned at a thickness of 5-μm. Section staining was performed as previously described [3]. All of the images were captured using an Olympus digital camera (Tokyo, Japan) and analyzed using Image-Pro Plus software (Media Cybernetics; Rockville, MD, USA).

### 2.13. Flow Cytometry

After the treatments, the cells were collected for subsequent flow cytometry (BD FACSCelesta, Minneapolis, MN, USA). The cells were washed twice with cold PBS buffer; then, they were resuspended at 1.0 × 10^7^ cell/mL in 1 × PBS buffer. They were washed once with 1mL of 1 × Perm/Wash Buffer working solution and incubated with 1mL of Fixation/Permeabilization solution at 4 °C for 40 min. Then, the cells were washed and resuspended with 1 × Perm/Wash Buffer. Finally, the cells were incubated with intracellular/intranuclear antibody dyes at 4 °C for 30 min and washed with PBS. The cells were centrifuged at 300× *g* for 5 min and resuspended in 400 µL of PBS. They were then analyzed by a flow cytometer (Celesta; BD; San Jose, CA, USA) within 3 h.

### 2.14. Statistical Analysis

All of the results are expressed as the mean ± SD. One-way ANOVA followed by Tukey’s post-hoc test as implemented in SPSS V22.0 (IBM, New York, NY, USA) was used for statistical analysis. Differences with a value of *p* < 0.05 were considered to be statistically significant.

## 3. Results

### 3.1. PDGF-BB Regulates RhoGDI1 Stability via the PDGF Receptor

**Figure 1:**
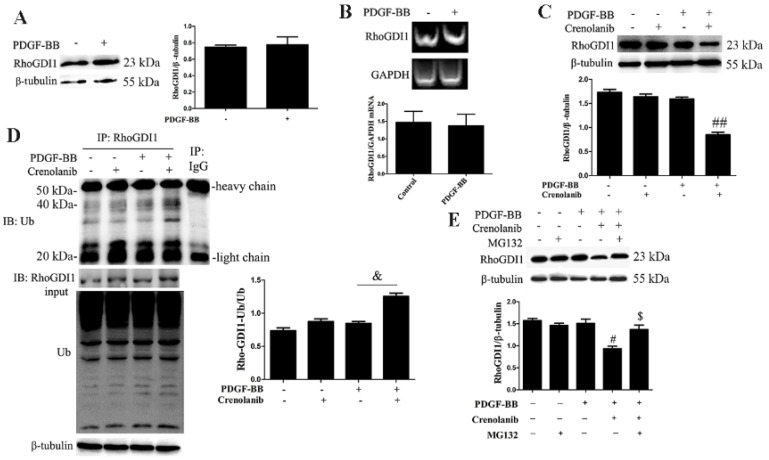
PDGF-BB stabilizes RhoGDI1 protein via the PDGF receptor. HA-VSMCs were pretreated with crenolanib (50 nM) for 48 h or MG132 (5 μM) for 1 h and then followed by 10 ng/mL PDGF-BB treatment for 24 h. Untreated cells were used as control. (**A**) Western blot analysis showed that PDGF-BB treatment did not affect RhoGDI1 protein level. Histogram showing the ratio of RhoGDI1 to β-tubulin (*n* = 3). (**B**) Real-time RT-PCR analysis also showed that PDGF-BB did not affect RhoGDI1 mRNA level. Histogram showing the ratio of RhoGDI1 mRNA level to GAPDH mRNA level (*n* = 3). (**C**) Western blot showed that crenolanib reduced RhoGDI1 protein level in PDGF-BB-treated HA-VSMCs. Histogram showing the ratio of RhoGDI1 to β-tubulin. ##, *p* < 0.01 vs. the PDGF-BB-treated group (*n* = 3). (**D**) Co-IP analysis of RhoGDI1 polyubiquitination. RhoGDI1 was immunoprecipitated from the cell lysates using the specific antibodies, and then the immunoprecipitated proteins were analyzed by western blotting. IgG immunoprecipitation was used as a negative control, showing heavy chain and light chain. Crenolanib treatment promoted RhoGDI1 polyubiquitination in PDGF-BB-induced cells. Histogram showing the ratio of RhoGDI1-binding ubiquitin to total ubiquitin. &, *p* < 0.05 vs. the PDGF-BB-treated group (*n* = 3). (**E**) Western blot showed that MG132 reversed the decreased RhoGDI1 protein level caused by crenolanib. Histogram showing the ratio of RhoGDI1 to β-tubulin. #, *p* < 0.05 vs. the PDGF-BB-treated group; $, *p* < 0.05 vs. the PDGF-BB and crenolanib-treated group (*n* = 3).

At present, the role of RhoGDI1 in PDGF-BB-induced VSMC phenotypic transformation is still unknown. To assess whether RhoGDI1 participates in the regulation of HA-VSMCs phenotypic transformation by PDGF-BB, we analyzed the protein and mRNA levels of RhoGDI1 by western blotting and real-time RT-PCR, respectively. As shown in Figure 1A,B, PDGF-BB treatment had no effect on the expression and transcription of RhoGDI1. Interestingly, crenolanib pretreatment significantly reduced the protein expression level of RhoGDI1 in PDGF-BB-induced HA-VSMCs (1.57 ± 0.38 vs. 0.81 ± 0.43, *p* < 0.01) (Figure 1C). We further investigated the effects of crenolanib on the polyubiquitination level of RhoGDI1 using Co-IP analysis and the effects of MG132 on the protein expression level of RhoGDI1 using western blotting. As shown in Figure 1D, crenolanib pretreatment significantly increased the ubiquitin levels of RhoGDI1 in PDGF-BB-mediated HA-VSMCs (0.85 ± 0.32 vs. 1.26 ± 0.58, *p* < 0.05). However, PDGF-BB treatment did not affect RhoGDI1 polyubiquitination compared with the control HA-VSMCs. In addition, MG132 pretreatment reversed the decreased protein expression level of RhoGDI1 caused by crenolanib (Figure 1E).

### 3.2. RhoGDI1 Protein Stability Promotes PDGF-BB-Induced VSMC Phenotypic Modulation

**Figure 2:**
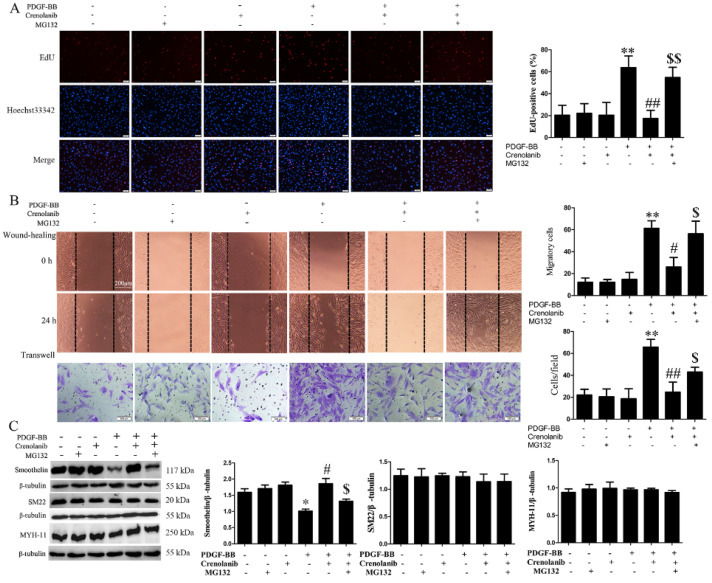
MG132 pretreatment blocks the reduced phenotypic transition of HA-VSMCs induced by crenolanib. HA-VSMCs were pretreated with crenolanib (50 nM) for 48 h or MG132 (5 μM) for 1 h and then exposed to 10 ng/mL PDGF-BB treatment for 24 h. Untreated cells were used as control. (**A**) Cell proliferation was checked by EdU assay (scale bar = 100 μm). Histogram showing the ratio of EdU-positive cells (red) to the total number of cells. **, *p* < 0.01 vs. the control group; ##, *p* < 0.01 vs. the group treated with PDGF-BB; $$, *p* < 0.01 vs. the PDGF-BB and crenolanib-treated group (*n* = 3). (**B**) Cell migration was checked by wound-healing (scale bar = 200 μm) and transwell assays (scale bar = 100 μm). Histograms showing the quantification of the wound healing (migrating cells from the scratched boundary) and transwell assay (crystal violet stained cells migrating to the lower chamber) results. **, *p* < 0.01 vs. the control group; #, *p* < 0.05 and ##, *p* < 0.01 vs. the PDGF-BB-treated group; $, *p* < 0.05 vs. the PDGF-BB and crenolanib-treated group (*n* = 3). These results show that MG132 pretreatment promoted the reduced cell proliferation and migration mediated by crenolanib. (**C**) Western blot analysis of MYH-11, SM22, and smoothelin. MG132 reduced the increased smoothelin expression induced by crenolanib. Histograms showing the ratios of target proteins to β-tubulin. *, *p* < 0.05 vs. the control group; #, *p* < 0.05 vs. the PDGF-BB-treated group; $, *p* < 0.05 vs. the PDGF-BB and crenolanib-treated group (*n* = 3).

PDGF-BB has previously been reported to induce VSMC proliferation and migration, which can be inhibited by crenolanib pretreatment [3]. In this study, we used EdU, wound healing, and transwell assays to investigate cell proliferation and migration. We found that crenolanib significantly inhibited PDGF-BB-induced cell proliferation and migration, which is consistent with the findings of previous reports (Figure 2A,B). In addition to VSMC proliferation and migration, the expression levels of contractile proteins, such as MYH11, SM22, and smoothelin, also serve as important indicators of VSMC phenotypic transformation. To investigate the effect of PDGF-receptor signaling on the expression of contractile proteins in HA-VSMCs, we measured the protein expression levels of MYH11, SM22, and smoothelin by western blotting. As shown in Figure 2C, PDGF-BB significantly decreased the expression of smoothelin, but the effect was reversed by crenolanib pretreatment. However, PDGF-receptor signaling exerted no significant effects on MYH11 and SM22 expression levels. Crenolanib treatment alone did not affect cell proliferation, migration, or the expression of contractile proteins. To further confirm the reduced expression of smoothelin in PDGF-treated HA-VSMCs, we checked intracellular smoothelin expression by flow cytometry. The results showed that PDGF-BB did reduce the expression of smoothelin in HA-VSMCs (Figure S1).

**Figure 3:**
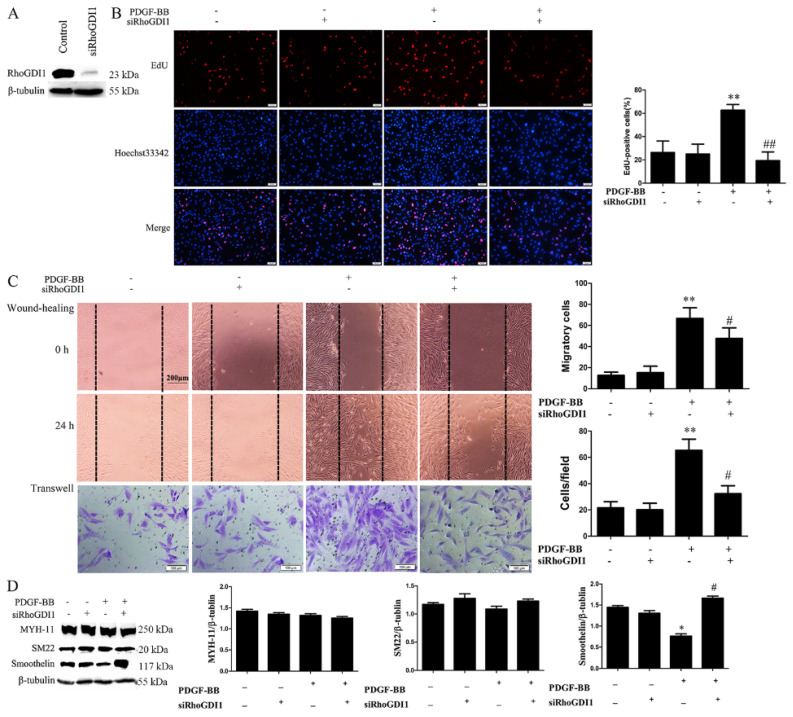
RhoGDI1 suppression inhibits PDGF-BB-induced phenotypic transition in HA-VSMCs. Cells were transfected with RhoGDI1 siRNA for 48 h and then treated with 10 ng/mL of PDGF-BB for another 24 h. Untreated cells were used as control. (**A**) Confirmation of RhoGDI1 knockdown by western blotting. (**B**) EdU assay showed that RhoGDI1 suppression inhibited PDGF-BB-induced cell proliferation (scale bar = 100 μm). Histogram showing the ratio of EdU-positive cells (red) to total cells. **, *p* < 0.01 vs. the control group; ##, *p* < 0.01 vs. the group treated with PDGF-BB (*n* = 3). (**C**) Wound-healing (scale bar = 200 μm) and transwell assays (scale bar = 100 μm) showed that RhoGDI1 knockdown reduced cell migration induced by PDGF-BB. The quantification method refers to Figure 2. **, *p* < 0.01 vs. the control group; #, *p* < 0.05 vs. the PDGF-BB-treated group (*n* = 3). (**D**) Western blot analysis of MYH-11, SM22, and smoothelin showed that RhoGDI1 suppression promoted the expression of smoothelin in PDGF-BB-treated cells. Histograms showing the ratios of target proteins to β-tubulin. *, *p* < 0.05 vs. the control group; #, *p* < 0.05 vs. the PDGF-BB-treated group (*n* = 3).

To further clarify the relationship between RhoGDI1 stability and VSMC phenotypic transformation, we analyzed the effects of MG132 pretreatment and RhoGDI1 knockdown on PDGF-BB-mediated VSMC phenotypic modulation. Figure 2A,B show that MG132 pretreatment significantly inhibited the reduced proliferation (15.32 ± 7.32 vs. 53.5 ± 7.25%, *p* < 0.01) and migration (wound healing, 24.22 ± 8.31 vs. 56.82 ± 9.66 cells, *p* < 0.05; transwell, 22.33 ± 8.37 vs. 41.78 ± 3.69 cells, *p* < 0.05) mediated by crenolanib in PDGF-BB-treated HA-VSMCs. In addition, MG132 pretreatment reduced the restored protein expression level of smoothelin induced by crenolanib (1.78 ± 0.13 vs. 1.28 ± 0.06, *p* < 0.05), without affecting MYH11 or SM22 expression (Figure 2C). Furthermore, we suppressed RhoGDI1 expression using siRNA (Figure 3A) and assessed the PDGF-BB-induced phenotypic transformation in HA-VSMCs. Figure 3B,C show that suppressing RhoGDI1 expression significantly inhibited PDGF-BB-induced cell proliferation (61.53 ± 4.42 vs. 19.23 ± 6.76%, *p* < 0.01) and migration (wound healing, 66.59 ± 8.33 vs. 47.13 ± 7.15 cells, *p* < 0.05; transwell, 65.42 ± 6.91 vs. 31.64 ± 4.96 cells, *p* < 0.05). The reduced expression of smoothelin induced by PDGF-BB was significantly inhibited by RhoGDI1 suppression (0.69 ± 0.04 vs. 1.53 ± 0.05, *p* < 0.05) (Figure 3D). However, there was no obvious change in MYH11 or SM22 expression after RhoGDI1 knockdown in PDGF-BB-treated HA-VSMCs. MG132 pretreatment or suppression of RhoGDI1 alone in the absence of PDGF-BB did not significantly affect cell phenotypic modulation.

### 3.3. PDGF-BB Promotes RhoGDI1-Cdc42 Interaction and Thus Cdc42 Activation by Its Receptor

**Figure 4:**
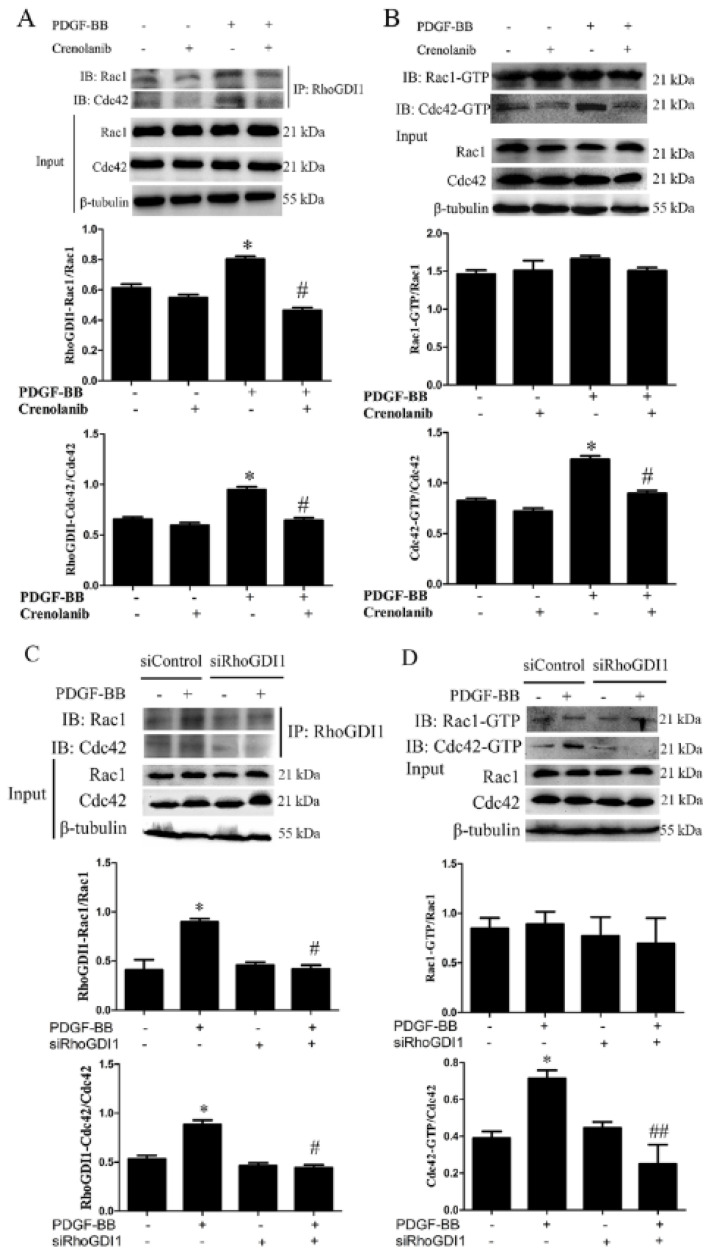
Both crenolanib pretreatment and RhoGDI1 suppression inhibit the activation of Cdc42 and its binding to RhoGDI1. HA-VSMCs were pretreated with crenolanib (50 nM) for 48 h, followed by treatment with 10 ng/mL of PDGF-BB for 24 h. The cells were transfected with control or RhoGDI1 siRNA for 48 h and then treated with 10 ng/mL of PDGF-BB for another 24 h. Untreated cells or cells transfected with control siRNA alone were used as control. (**A** and **C**) Analysis of the interaction of RhoGDI1 with Rac1 or Cdc42 using Co-IP analysis. RhoGDI1 was immunoprecipitated from the cell lysates using the specific antibodies, and then the immunoprecipitated proteins were analyzed by western blotting. Crenolanib pretreatment and RhoGDI1 suppression inhibited the binding of RhoGDI1 with Rac1 or Cdc42 induced by PDGF-BB. Histograms showing the ratios of RhoGDI1-binding Rac1 or Cdc42 to total Rac1 or Cdc42. *, *p* < 0.05 vs. the control group; #, *p* < 0.05 vs. the PDGF-BB-treated group (*n* = 3). (**B** and **D**) Detection of Rho GTPases activity by pulldown assay. Crenolanib pretreatment and RhoGDI1 suppression inhibited PDGF-BB-induced activation of Cdc42 without affecting Rac1 activation. Histograms showing the ratios of Rac1 or Cdc42 GTP-bound to total Rac1 or Cdc42. *, *p* < 0.05 vs. the control group; #, *p* < 0.05 and ##, *p* < 0.01 vs. the PDGF-BB-treated group (*n* = 3).

To investigate the effects of PDGF-receptor signaling on the interaction between RhoGDI1 and Rac1 or Cdc42 in HA-VSMCs, we measured the interaction of RhoGDI1-and Rac1 or -Cdc42 using Co-IP analysis and the activation of Rac1 or Cdc42 using pulldown analysis after crenolanib pretreatment. Figure 4A shows that PDGF-BB treatment significantly increased the interaction of RhoGDI1 with both Rac1 and Cdc42; however, the effect was obviously reduced by crenolanib pretreatment. However, as illustrated in Figure 4B, in response to PDGF-BB treatment for 24 h, there was an improvement in the activation of Cdc42 (0.78 ± 0.03 vs. 1.21 ± 0.05, *p* < 0.05) but not Rac1. Although some studies have reported that PDGF-BB can induce Rac1 activation in different in vitro models [29], our study showed that Rac1 was transiently activated between 0 and 24 h and then returned to the normal level at 12 h (Figure S2). The increased activation of Cdc42 induced by PDGF-BB was significantly inhibited by crenolanib (1.21 ± 0.05 vs. 0.81 ± 0.04, *p* < 0.05). To further clarify the effect of the RhoGDI1-Rho GTPase interaction on the activation of Rho GTPases, we reduced the interaction of RhoGDI1 with Rho GTPases in PDGF-BB-treated cells by knocking down RhoGDI1 (Figure 4C). As shown in Figure 4D, RhoGDI1 suppression decreased PDGF-BB-induced Cdc42 activation without affecting Rac1 activity (0.71 ± 0.04 vs. 0.25 ± 0.10, *p* < 0.01).

### 3.4. Inhibition of PDGF- RhoGDI1 Signaling Leads to the Accumulation of Cdc42 in the ER

**Figure 5:**
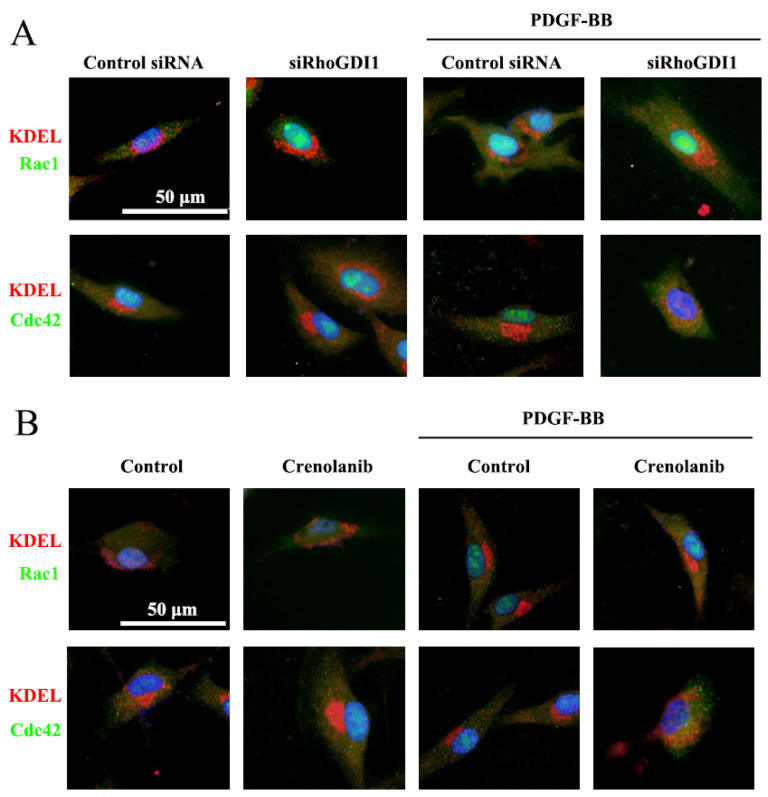
RhoGDI1 suppression (**A**) and crenolanib pretreatment (**B**) promotes the aggregation of Cdc42 in the endoplasmic reticulum (ER) in PDGF-BB-treated HA-VSMCs. scale bar = 50 μm. (**A**,**B**) Representative images of Rac1 or Cdc42 (green) and ER (KDEL, red) as double staining (merge). Nuclei were stained with DAPI (blue). (**A**) HA-VSMCs transfected with control siRNA and RhoGDI1 siRNA were treated with 10 ng/mL of PDGF-BB for 24 h. The cells transfected with control siRNA alone were used as control. RhoGDI1 knockdown enhanced the co-localization of the ER and Cdc42 but did not affect the co-localization of the ER and Rac1 in PDGF-BB-treated HA-VSMCs. (**B**) HA-VSMCs were treated with crenolanib (50 nM) for 48 h followed by 10 ng/mL of PDGF-BB for 24 h. Untreated cells were used as control. Crenolanib pretreatment enhanced the co-localization of the ER and Cdc42 but did not affect the co-localization of the ER and Rac1 in PDGF-BB-treated HA-VSMCs.

To confirm the effect of RhoGDI1 on the regulation of Rho GTPase location, we assessed the accumulation of Rho GTPases in the ER after RhoGDI1 knockdown using immunofluorescence double staining. As shown in Figure 5A, in response to PDGF-BB treatment, there was no obvious co-localization of Rac1 or Cdc42 with the ER by staining the ER (ER marker KDEL) red and Rho GTPases green. Similarly, the co-localization of Rac1 or Cdc42 with the ER was not observed in the control (control siRNA) and RhoGDI1 suppression group without PDGF-BB treatment. RhoGDI1 deletion significantly promoted the co-localization of Cdc42 with the ER and the accumulation of Cdc42 in the ER in PDGF-BB-treated HA-VSMCs. Meanwhile, it did not affect the co-localization of Rac1 and ER and did not promote the aggregation of Rac1 in the ER in PDGF-BB-treated cells. We next investigated the effect of crenolanib on the co-localization of Rac1 or Cdc42 with the ER. Similarly to the results in Figure 5A, Figure 5B shows that crenolanib pretreatment stimulated the co-localization of Cdc42 with the ER in PDGF-BB-treated cells without affecting Rac1-ER co-localization. Rac1 or Cdc42 and the ER were not significantly co-localized in the group without treatment or in the group treated with crenolanib alone.

### 3.5. Cdc42 Activation Participates in PDGF-BB-Induced VSMC Phenotypic Modulation

**Figure 6:**
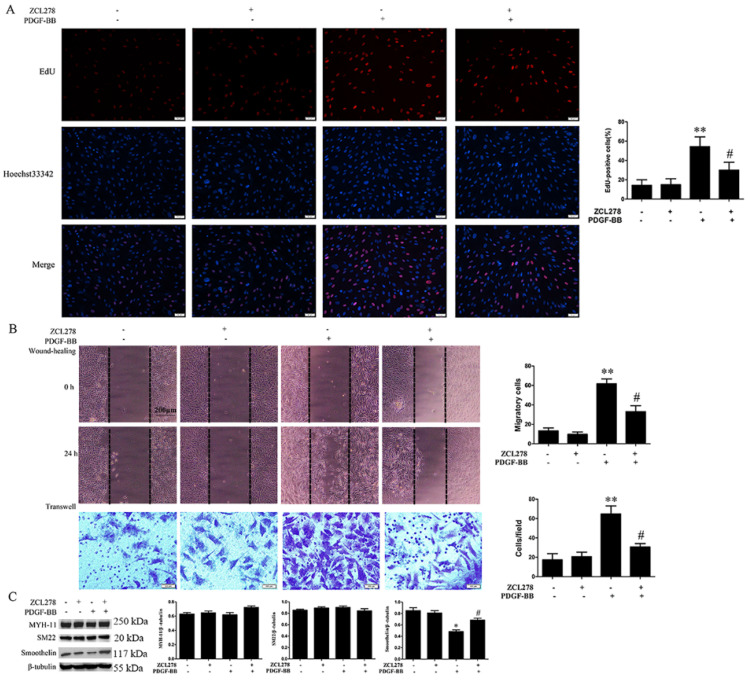
Cdc42 inhibition reduces PDGF-BB-induced phenotypic transition in HA-VSMCs. HA-VSMCs were pretreated with ZCL278 (50 μM) for 30 min followed by treatment with 10 ng/mL of PDGF-BB for 24 h. Untreated cells were used as control. (**A**) Cell proliferation was analyzed by EdU assay (scale bar = 100 μm). Histogram showing the ratio of EdU-positive cells (red) to total cells. **, *p* < 0.01 vs. the control group; #, *p* < 0.05 vs. the PDGF-BB-treated group (*n* = 3). (**B**) Cell migration was analyzed by wound-healing (scale bar = 200 μm) and transwell assays (scale bar = 100 μm). The quantification method refers to Figure 2. **, *p* < 0.01 vs. the control group; #, *p* < 0.05 vs. the PDGF-BB-treated group (*n* = 3). ZCL278 pretreatment significantly decreased cell proliferation and migration induced by PDGF-BB. (**C**) Western blot analysis of MYH-11, SM22, and smoothelin. ZCL278 pretreatment increased the expression of smoothelin in PDGF-BB-treated cells. Histograms showing the ratios of the target proteins to β-tubulin. *, *p* < 0.05 vs. the control group; #, *p* < 0.05 vs. the PDGF-BB-treated group (*n* = 3).

To verify whether Rac1 or Cdc42 activation is associated with PDGF-BB-induced VSMC phenotypic transformation, we assessed the effects of Rac1 or Cdc42 inhibition on VSMC transformation. Figure S3A,B show that the Rac1 inhibitor (NSC23766) did not affect HA-VSMC proliferation and migration, which were mediated by PDGF-BB. In addition, NSC23766 pretreatment did not affect the reduced expression of smoothelin induced by PDGF-BB (Figure S3C). However, Cdc42 inhibition (ZCL278 pretreatment) significantly reduced cell proliferation (53.23 ± 7.53 vs. 28.41 ± 6.12%, *p* < 0.05) and migration (wound healing, 61.37 ± 4.89 vs. 31.03 ± 5.13 cells, *p* < 0.05; transwell, 62.83 ± 6.85 vs. 28.75 ± 3.21 cells, *p* < 0.05) and promoted the expression of smoothelin (0.47 ± 0.04 vs. 0.68 ± 0.05, *p* < 0.05) in PDGF-BB-treated HA-VSMCs (Figure 6).

### 3.6. RhoGDI1-Cdc42 Signaling Participates in VSMC Phenotypic Transformation and Neointima Formation in Rats.

**Figure 7:**
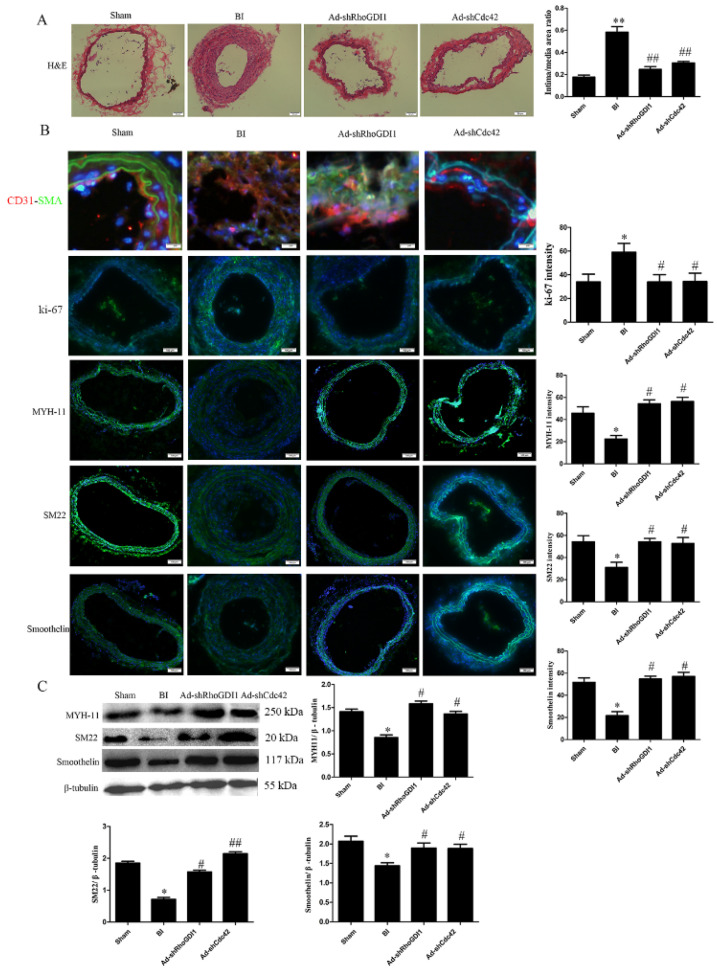
Both RhoGDI1 and Cdc42 suppression inhibit neointima formation and improve the expression of contractile proteins in a rat carotid injury model. For shRNA-mediated knockdown, the carotid artery was injected with approximately 0.2 mL of virus solution (titer: 1 × 10^10^ pfu) after balloon injury operation. (**A**) H&E of arteries 14 days after balloon injury. Rats without balloon injury were used as the sham operation group. Histogram showing the area ratio of intima to media. Both RhoGDI1 and Cdc42 knockdown reduced intima/media area ratio. **, *p* < 0.01 vs. the sham operation group; ##, *p* < 0.01 vs. the injury model group (*n* = 10). (**B**) Immunofluorescence staining for contractile proteins such as MYH-11, SM22, smoothelin (green), ki-67 (green), and α-SMA (green) and CD31 (red) as double staining (merge); nuclei were stained with DAPI (blue). Histograms showing the fluorescence intensity of the staining. RhoGDI1 and Cdc42 knockdown reduced the expression of ki-67 and co-localization of α-SMA with CD31 and increased the expression of the three contractile proteins. *, *p* < 0.05 vs. the sham operation group; #, *p* < 0.05 vs. the injury model group (*n* = 10). (**C**) Western blots showing the expression of MYH-11, SM22, and smoothelin. Histograms showing the ratios of the contractile proteins to β-tubulin. The results are consistent with those from immunofluorescence analysis. *, *p* < 0.05 vs. the sham operation group; #, *p* < 0.05 and ##, *p* < 0.01 vs. the injury model group (*n* = 10).

We verified the effects of RhoGDI1 and Cdc42 suppression on VSMC phenotypic transformation and neointima formation in vivo using a rat BI model. The knockdown of RhoGDI1 or Cdc42 in rats was confirmed by western blot analysis (Figure S4). Figure 7A shows that BI resulted in significant neointima formation, as assessed by quantification of the intima/media area ratio (0.18 ± 0.02 vs. 0.59 ± 0.05, *p* < 0.01). Knockdown of either RhoGDI1 or Cdc42 significantly reduced neointima formation, as evidenced by 63% and 60% decreases in the intima/media area ratio, respectively. To investigate whether RhoGDI-Cdc42 signaling participates in VSMC phenotypic modulation, we also examined the expression of contractile proteins, ki-67, and co-localization of CD31 (endothelial marker) and α -SMA (myofiber marker) by immunostaining and western blotting. BI significantly induced ki-67 expression and co-localization of α-SMA with CD31 in neointima, suggesting that VSMCs proliferated and migrated into the neointima in the rat BI model. Knockdown of RhoGDI1 or Cdc42 reduced the expression of ki-67 and co-localization of α-SMA with CD31 after BI in the rats (Figure 7B), which confirms that the RhoGDI1-Cdc42 signaling is required for VSMC proliferation and migration. These results are consistent with those from the in vitro experiments with HA-VSMCs. However, dissimilar to those in HA-VSMCs, the expression levels of MYH11, SM22 and smoothelin were significantly downregulated in rat carotid arteries after BI. Knockdown of RhoGDI1 and Cdc42 promoted the expression of these contractile markers (Figure 7B,C).

## 4. Discussion

VSMCs expressing contractile marker genes play key roles in regulating blood vessel contraction. They can switch from the contractile phenotype to the proliferative and migratory phenotype under stimulation by factors such as PDGF-BB, insulin-like growth factors (IGFs), and interferon gamma (IFN-γ) [30,31,32]. It has been reported that RhoGDI1 expression plays crucial roles in various tumor cellular functions such as proliferation and migration, as well as a tumor’s biological behaviors [15,16,17,18,19,20]. In addition, RhoGDI1 knockout promotes endothelial permeability in mice [33]. There have been a few reports on the roles of RhoGDI1 in regulating the functions of VSMCs. We have previously reported that RhoGDI participates in hypertensive vascular remodeling that is activated by the RAS [21]. However, the effect of RhoGDI1 on traumatic intimal hyperplasia and the mechanisms behind these effects are not well understood.

Considering that PDGF-BB secretion increases during neointima formation [3], we used a PDGF-BB-induced VSMC proliferation and migration model in vitro. Our results demonstrated that PDGF-BB promoted RhoGDI1 stability via the PDGF receptor, but not its expression (Figure 1). Inhibition of the PDGF receptor significantly increased RhoGDI1 polyubiquitination, which then led to RhoGDI1 degradation by proteasomes (Figure 1). Therefore, we suggest that PDGF receptor signaling participates in the regulation of RhoGDI1 protein degradation or stability. In this study, we only analyzed the polyubiquitination of RhoGDI1, given that ubiquitination modification is closely related to protein stability, although many reports have indicated that RhoGDI1 can be regulated by SUMOylation and isoprenylation as well [34,35]. We will continue to explore other post-translational modifications of RhoGDI1 and their effects on RhoGDI1 function in our follow-up work. Furthermore, inhibition of RhoGDI1 degradation by MG132 significantly promoted cell proliferation and migration and reduced the expression of the contractile protein smoothelin in PDGF-BB-induced HA-VSMCs (Figure 2). In addition, RhoGDI1 knockdown inhibited HA-VSMC proliferation and migration, and it increased smoothelin expression (Figure 3), suggesting that RhoGDI1 stability affects PDGF-BB-induced HA-VSMC phenotypic modulation. These findings indicate that PDGF-receptor signaling maintains the stability of RhoGDI1 and thus causes HA-VSMC phenotypic transformation which can be prevented by RhoGDI1 degradation.

In the current study, RhoGDI1 knockdown also alleviated neointima formation in the model of rat traumatic intimal hyperplasia (Figure 7A). According to our findings, RhoGDI1 participates in VSMC proliferation and migration from the media to neointima after BI. Importantly, rat BI reduced the expression of three contractile proteins—including MYH-11, SM22, and smoothelin (Figure 7B,C)—although PDGF-BB only reduced the expression of smoothelin in vitro (Figure 2C). This observation may reflect the different origin of the cells in vitro and in vivo (from different species) or increased secretion of factors other than PDGF-BB in the BI model. Smoothelin expression may be more sensitive in PDGF-mediated phenotypic transformation in human cell lines than in rats, or the BI may induce signals other than PDGF-BB signaling—such as PI3K/Akt and MAPK/ERK signaling— thereby stimulating changes in the expression of MYH-11 and SM22 [36,37]. Knockdown of RhoGDI1 significantly increased the expression of these three contractile proteins, which may help rat VSMCs to maintain their contractile phenotype (Figure 7B,C).

RhoGDIs have been shown to regulate Rho-family GTPase stability, activation, and the crosstalk between Rho GTPases [14,15,16,38]. The small Rho GTPase family of proteins— including the three major G-protein classes RhoA, Rac1 and Cdc42—plays crucial roles in regulating multiple common cellular functions [23,24]. We have previously reported that the RhoA/ROCK signaling regulates smooth muscle phenotypic modulation and vascular remodeling via the JNK pathway and vimentin cytoskeletal network [3]. In addition, it has been reported that the cooperation between PKA, RhoA, and an RhoGDI forms a pacemaker that governs the morphodynamic behavior of migrating cells [39]. Cdc42 activation has been reported to participate in the polarization and sprouting of endothelial cells and in vascular angiogenesis [40,41]. Cdc42 deficiency can delay endothelial migration [42,43]. However, prior to this study, the regulatory roles of RhoGDI1 on Rac1 or Cdc42 in VSMC phenotypic transformation and neointima formation have not been clarified. Our study was the first to demonstrate that PDGF-BB promotes the interaction of RhoGDI1 with Rac1 or Cdc42 and the activation of Cdc42 via the PDGF receptor (Figure 4). It has been reported that PDGF promotes the dissociation of RhoGDI from Rac1 and Cdc42; however, the results are based on an in vitro model involving PDGF treatment of HeLa cells for 10 min [44], which is completely different from our current in vitro model of PDGF-treated HA-VSMCs for 24 h. The inhibition of the RhoGDI-Cdc42 interaction via RhoGDI knockdown significantly decreased the activation of Cdc42 (Figure 4D), suggesting that Cdc42 activation is associated with PDGF-receptor signaling and the interaction with RhoGDI1. Interestingly, Rac1 was transiently activated under PDGF-BB treatment, and then returned to normal levels at 12 h (Figure S2). In this study, PDGF-BB treatment for 24 h did not induce Rac1 activation, and the activation of Rac1 after 24 h treatment with PDGF-BB was not affected by the PDGF-BB signaling and the RhoGDI1-Rac1 interaction (Figure 4). However, it has been reported that Rac1 activity is controlled by guanine nucleotide exchange factors (GEFs) for activation, GTPase-activating protein (GAPs, such as β2-chimaerin) for inactivation, and RhoGDIs for spatiotemporal regulation [45,46]. In our model, we speculate that Rac1 activation may be mainly regulated by GEFs or β2-chimaerin signals, which are not affected by the RhoGDI1 pathway.

RhoGDI1 facilitates the localization and activation of Rho GTPase to the PM; otherwise, Rho GTPase will be located in the ER [15]. In the current study, we demonstrated that PDGF signaling inhibition and RhoGDI1 reduction promoted the accumulation of Cdc42 in the ER without affecting the intracellular distribution of Rac1 (Figure 5). This result suggests that the PDGF-RhoGDI1 signaling participates in the cellular localization of Cdc42, and PDGF-RhoGDI1 signaling suppression hinders Cdc42 from leaving the ER, which makes it difficult to locate and activate Cdc42 in the PM. Therefore, we hypothesize that the PDGF-RhoGDI1 pathway can facilitate the localization and activation of Cdc42 to the PM. In addition, the inhibition of Cdc42 rather than Rac1 reduced PDGF-BB-induced VSMC phenotype transformation (Figure 6 and Figure S3). This indicates that Cdc42 activation, but not Rac1 activation, participates in PDGF-BB-induced VSMC phenotypic modulation. Furthermore, both RhoGDI1 and Cdc42 knockdown significantly reduced neointima formation, ki-67 expression, and α-SMA-CD31 co-localization, and increased contractile protein expression in the model of rat traumatic intimal hyperplasia (Figure 7). These results indicate that the RhoGDI1-Cdc42 signaling participates in VSMC phenotypic transformation and pathological intimal hyperplasia.

## 5. Conclusions

In summary, the main findings of this study are as follows: (1) RhoGDI1 stability, which is regulated by the PDGF receptor, participates in VSMC phenotypic transformation and neointima formation; (2) RhoGDI1 stability promotes the interaction of RhoGDI1 and Cdc42 and then facilitates the localization and activation of Cdc42 to the PM, whereas Rac1 subcellular localization and activity are regulated by an unknown mechanism independent of RhoGDI1 in PDGF-BB-induced VSMCs; (3) the activation of Cdc42, but not Rac1, plays an important role in VSMC phenotypic transformation and neointima formation; (4) the inhibition of the PDGF signaling promotes the ubiquitination and degradation of RhoGDI1 and the accumulation of Cdc42 in the ER, thereby inhibiting Cdc42 activation and VSMC transformation. It should be noted that our findings are more specific for traumatic neointima formation, such as in the rat BI model.

## Data Availability

The data presented in this study are available upon request from the corresponding author.

## Supplementary Materials

The following are available online at https://www.mdpi.com/article/10.3390/biomedicines9091169/s1, Figure S1: PDGF-BB reduced the intracellular expression of smoothelin in HA-VSMCs. Figure S2: Rac1 has a transient activation between 0 and 24 h treatment of PDGF-BB. Figure S3: Rac1 inhibition does not affect PDGF-BB-induced phenotypic transition in HA-VSMCs. Figure S4: The confirmation of RhoGDI1 and Cdc42 knockdown in rats by western blot.
