# Supplementary material for: RhoGDI1-Cdc42 Signaling Is Required for PDGF-BB-Induced Phenotypic Transformation of Vascular Smooth Muscle Cells and Neointima Formation
Source: Biomedicines. 2021 Sep 6;9(9):1169. doi: 10.3390/biomedicines9091169 (PMC8470270; doi:10.3390/biomedicines9091169)
Supplement: Supplementary file 1 [file biomedicines-09-01169-s001.zip › biomedicines-1313392-supplementary.pdf]

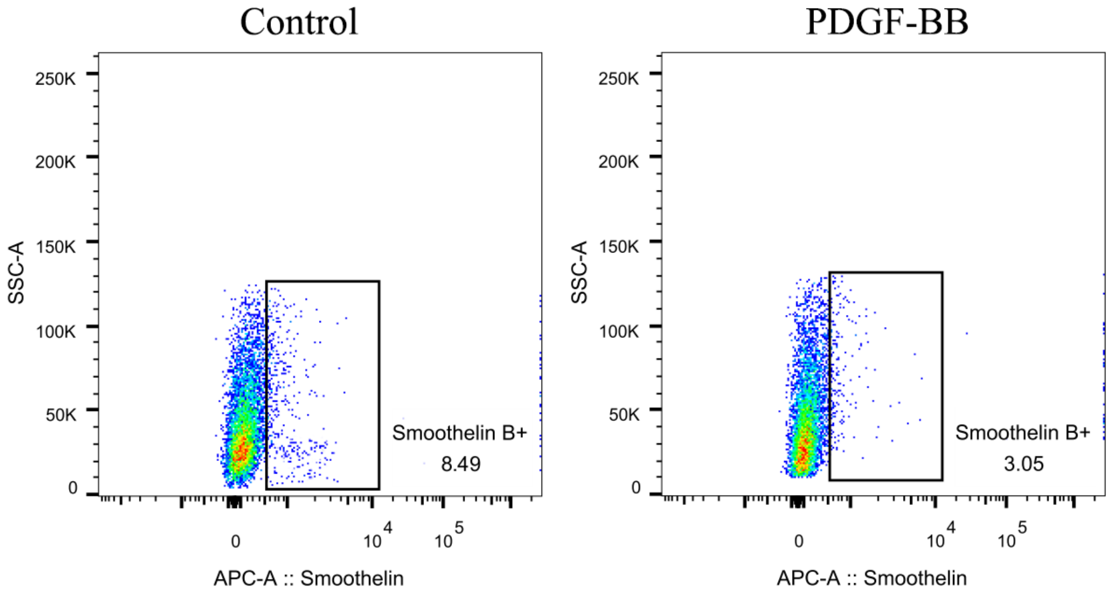

Fig. S1

Figure S1 PDGF-BB reduced the intracellular expression of smoothelin in HA-VSMCs. HA-VSMCs were treated with 10 ng/mL PDGF-BB for 24 h. Untreated cells were used as control. Cells were incubated with intracellular/intranuclear antibody dyes (AF647-labeled smoothelin antibody) at 4°C for 30 min and then checked by the flow cytometer. The ratio of the number of positive cells expressing smoothelin in the normal group and the PDGF-BB group was 8.49/3.05; this indicates that PDGF-BB significantly reduced the intracellular expression of smoothelin in HA-VSMCs, which is consistent with the results of western blotting.

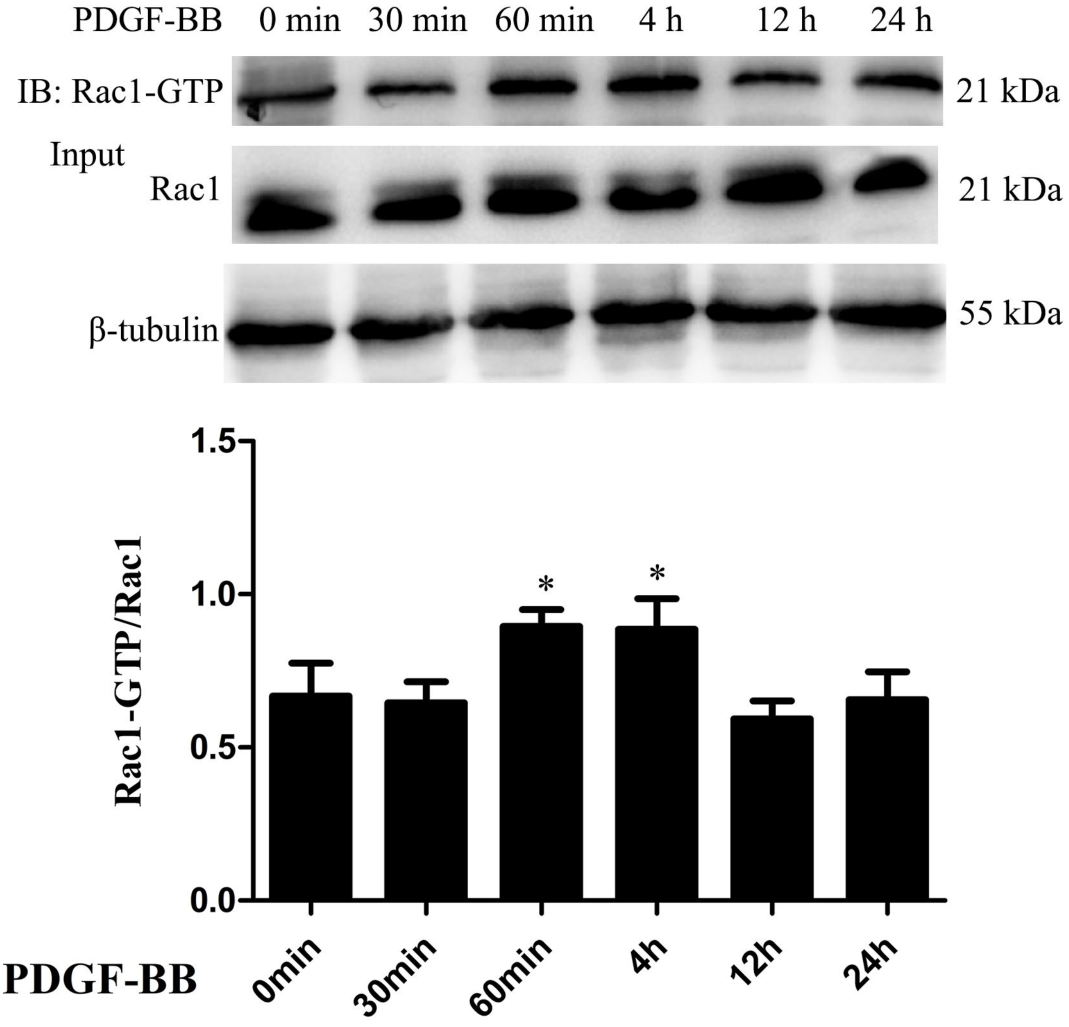

Fig. S2

Figure S2 Rac1 has a transient activation between 0 and 24 h treatment of PDGF-BB. HA-VSMCs were treated with 10 ng/mL PDGF-BB at different time points (0min, 30min, 60min, 4h, 12h, 24h). Cells without PDGF-BB treatment were used as control. Pulldown assay was used to check Rac1 activity. Rac1 activity had a transient increase at 60min and 4h of PDGF-BB treatment, and then returned to the normal level at 12 h. Histogram showing the ratio of Rac1 GTP-bound to total Rac1. *, $p < 0.05$ vs. the control group.

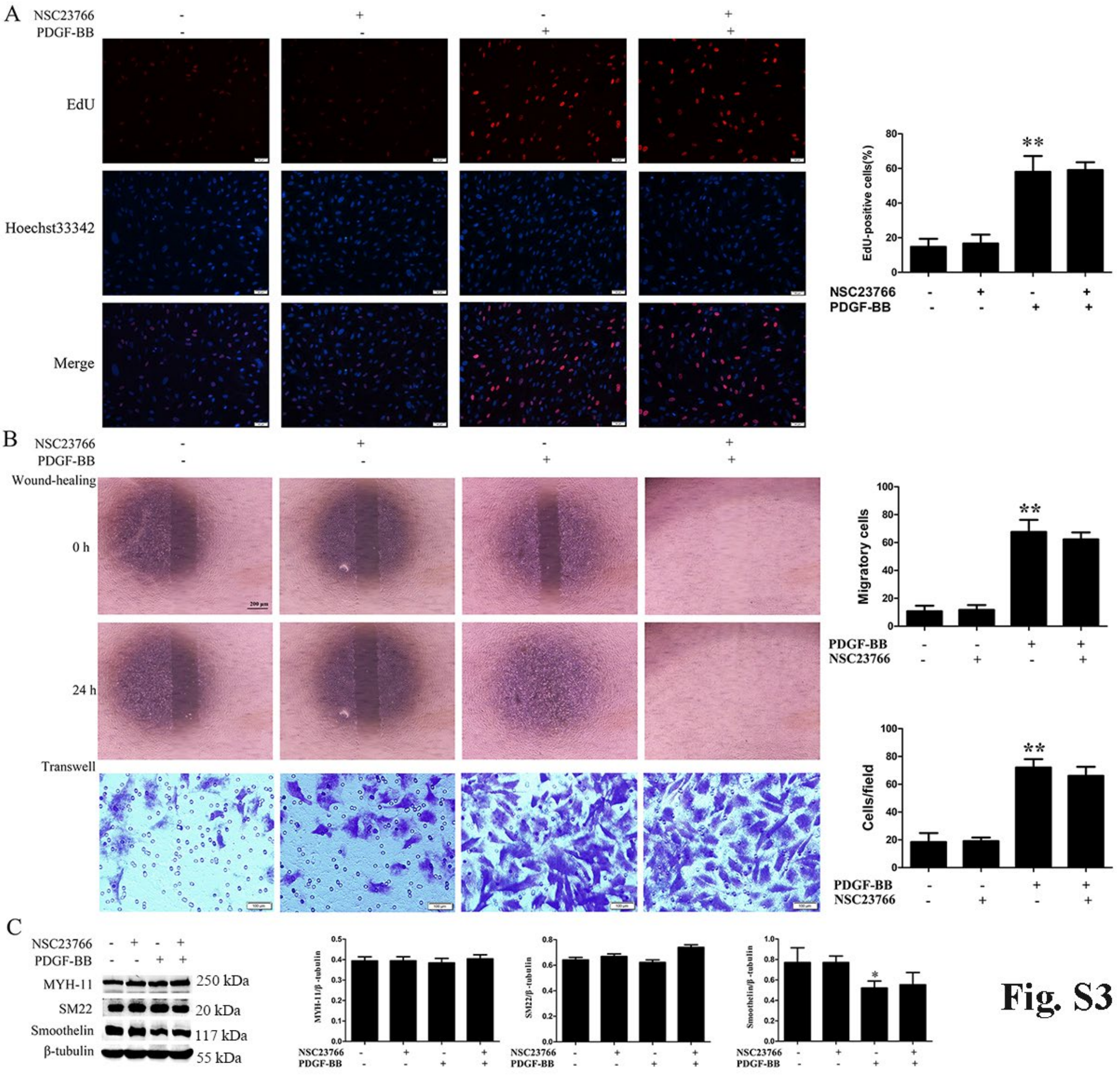

Figure S3 Rac1 inhibition does not affect PDGF-BB-induced phenotypic transition in HA-VSMCs. HA-VSMCs were pretreated with NSC23766 (50 μM) for 30min followed by 10 ng/mL PDGF-BB for 24 h. Untreated cells were used as control. (A) Cell proliferation was checked by EdU assay. Histogram showing the ratio of EdU-positive cells (red) to total cells. **, $p < 0.01$ *vs.* the control group (n =3). (B) Cell migration was checked by wound-healing and Transwell assays. Histograms showing the quantification of the wound healing and Transwell assay results. **, $p < 0.01$ *vs.* the control group (n =3). NSC23766 pretreatment did not affect both cell proliferation and migration induced by PDGF-BB. (C) Western blot analysis of MYH-11, SM22, and smoothelin. It showed that NSC23766 pretreatment did not affect the expression of smoothelin in PDGF-BB-treated cells. Histograms showing the ratios of target proteins to β-tubulin. *, $p < 0.05$ *vs.* the control group (n =3).

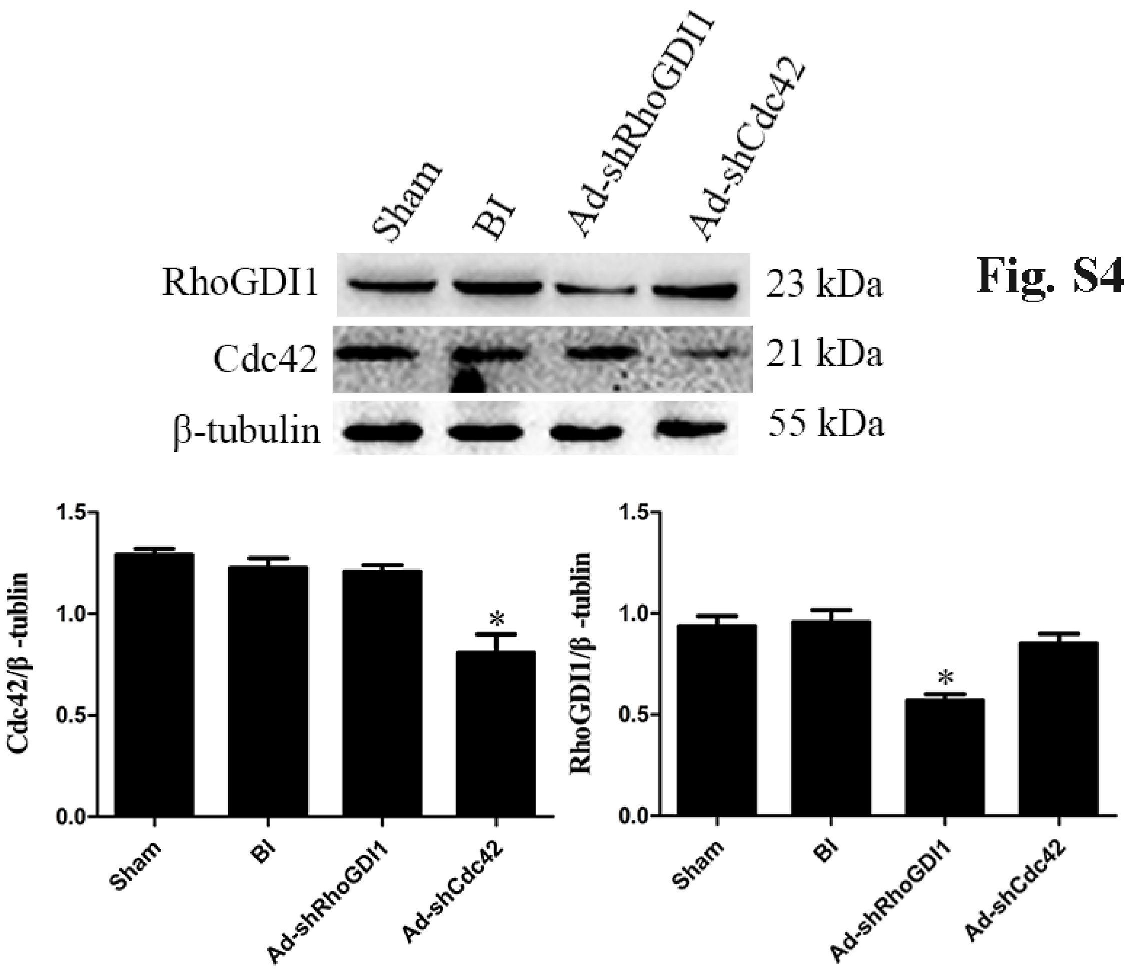

Figure S4 The confirmation of RhoGDI1 and Cdc42 knockdown in rats by western blot. Rat carotid artery was injected with 0.2 mL of virus solution after BI operation to knockdown the target gene. Rats without BI were used as the sham operation group. The expression of RhoGDI1 and Cdc42 was checked by western blot. It showed that RhoGDI1 and Cdc42 were successfully knocked down. Histograms showing the ratios of target proteins to β-tubulin. *, $p < 0.05$ vs. the sham operation group (n =10).
